# Prevalence and Risk Factors of Vitamin B_12_ Deficiency among Pregnant Women in Rural Bangladesh

**DOI:** 10.3390/nu14101993

**Published:** 2022-05-10

**Authors:** Odunayo Ifeoluwa Sobowale, Moududur Rahman Khan, Anjan Kumar Roy, Rubhana Raqib, Faruk Ahmed

**Affiliations:** 1 State Specialist Hospital, Osogbo 23022, Nigeria; dunifex@yahoo.com; 2 Institute of Nutrition and Food Science, University of Dhaka, Dhaka 1000, Bangladesh; khan.moudud@gmail.com; 3 International Centre for Diarrhoeal Disease Research, Mohakhali, Dhaka 1212, Bangladesh; anjan@icddrb.org (A.K.R.); rubhana@icddrb.org (R.R.); 4 Public Health, School of Medicine and Dentistry, Gold Coast Campus, Griffith University, Gold Coast, QLD 4220, Australia

**Keywords:** vitamin B_12_ deficiency, marginal vitamin B_12_ deficiency, pregnant women, Bangladesh

## Abstract

Vitamin B_12_ deficiency is associated with an increased risk of pregnancy complications and adverse birth outcomes. However, data on vitamin B_12_ deficiency in pregnant Bangladeshi women are limited. This study examines vitamin B_12_ deficiency and marginal deficiency in rural Bangladeshi women during early and late pregnancies. Some 522 women whose gestational age was <20 weeks were recruited. Serum vitamin B_12_ concentrations were measured at baseline and after 14 weeks of iron-folate supplementation. Logistic regression analysis examined the association of various socio-demographic, dietary, and pregnancy-related factors with vitamin B_12_ deficiency and marginal deficiency. Overall, 19% of the women during early pregnancy had vitamin B_12_ deficiency (serum vitamin B_12_ concentration < 203 pg/mL) and nearly 40% had marginal deficiency (serum vitamin B_12_ concentration 203 to <300 pg/mL). Vitamin B_12_ deficiency doubled to 38% during late pregnancy, while marginal deficiency slightly increased to 41.7%. The pregnant women with a gestational age of ≥27 weeks had a higher risk of developing vitamin B_12_ deficiency (OR = 2.61; 95% CI = 1.096–6.214) than those of a gestational age of <27 weeks. Vitamin B_12_ deficiency was significantly higher in pregnant women in rented accommodation (OR = 13.32; 95% CI = 1.55–114.25) than in those living in their own house. Vitamin B_12_ deficiency was significantly higher among women who consumed red or organ meat <3 times a week than in those who consumed it more often (OR = 2.327, 95% CI = 1.194–4.536). None of these factors were significantly associated with marginal vitamin B_12_ deficiency. In conclusion, vitamin B_12_ deficiency and marginal deficiency among pregnant rural Bangladeshi women increased as their pregnancies progressed. Increasing gestational age, living in a rented house, and the consumption of red or organ meat <3 times a week were identified as the independent risk factors of vitamin B_12_ deficiency in this population. Further research with more in-depth assessments of dietary vitamin B_12_ intakes is needed to develop an intervention program preventing vitamin B_12_ deficiency in this population.

## 1. Introduction

A deficiency in vitamin B_12_ (also known as cobalamin) has been identified as a significant public health problem globally [1,2]. While an impaired vitamin B_12_ status can occur in individuals of all ages, pregnant women, particularly those in less industrialized countries, are at higher risk of developing vitamin B_12_ deficiency [3,4,5].

Vitamin B_12,_ an essential micronutrient in the human body, is required for the synthesis of DNA, RNA, phospholipids, and neurotransmitters [3,6]. It also helps to catabolize branched-chain and odd-chain fatty acids [3,6,7]. Thus, vitamin B_12_ is crucial for cellular growth, differentiation, and development [8]. During pregnancy, women are more vulnerable to a deficiency of vitamin B_12_ due to increased metabolic demands imposed by physiological activities, such as the growth of the placenta, fetus, and maternal tissue [9]. Several studies have reported that the prevalence of vitamin B_12_ deficiency increases as pregnancy progresses, with the highest prevalence during the third trimester [4,10,11,12,13].

Vitamin B_12_ deficiency during pregnancy is associated with an increased risk of pregnancy complications, including spontaneous abortion [14], recurrent pregnancy loss [15,16], intrauterine growth restriction [17], low birth weight [17], and neural tube defects [5,18,19,20]. In a review of maternal vitamin B_12_ status and perinatal health, Finkelstein et al. [3] reported a significant association between maternal and neonatal vitamin B_12_ status at delivery. In a recent systematic review, Behere et al. [5] demonstrated an association between impaired maternal vitamin B_12_ status and adverse longer-term health outcomes, such as cognitive functions, adiposity, and insulin resistance in children.

Although vitamin B_12_ deficiency can be caused by malabsorption of food, the inadequate intake of animal-source foods is the main cause [21]. People who live in countries with restrictions on animal food consumption are susceptible to vitamin B_12_ deficiency. These restrictions could result from cultural and religious practices, or because these countries have a low socioeconomic status, and the consumption of animal-source foods is limited because of cost, lack of availability, or poor access to fortified foods or supplements [1,2,22,23]. Individuals adhering to a vegetarian diet are at higher risk of developing vitamin B_12_ deficiency [24], as their diet is predominantly plant-based, and the only food source rich in vitamin B_12_ comes from animal products [25]. Vitamin B_12_ deficiency is also caused by the malabsorption of food due to gastrointestinal infections, and nonspecific gastritis, including atrophy of the gastric mucosa, and gradual loss of gastric acid, particularly in the elderly [2,24].

In Bangladesh, some studies have revealed a high prevalence of vitamin B_12_ deficiency among pregnant women [26,27,28]. However, these studies have used either a very small sample size (*n* = 68) [28], or have only focused on early pregnancy. In addition, to date, no studies have examined the changes in vitamin B_12_ status with the progress of pregnancy. This is important as the requirement for vitamin B_12_ significantly increases in late pregnancy when fetal growth is intense. Of note, one in five children in Bangladesh are born with a low birth weight [29]. Furthermore, none of the previous studies in Bangladesh has reported the risk factors of vitamin B_12_ deficiency during pregnancy. Given the above limitations, and considering the importance of vitamin B_12_ during pregnancy, this research aims to determine the prevalence of vitamin B_12_ deficiency, and the associated risk factors of this deficiency, among rural Bangladeshi women during early and late pregnancy.

## 2. Materials and Methods

### 2.1. Study Population

The study group comprised 522 pregnant rural women with a gestational age of ≤20 weeks (defined as early pregnancy) in Bangladesh. This longitudinal study was carried out between April and October 2015 while collecting data for an intervention study examining the effect of routine iron-folic acid supplementation during pregnancy.

### 2.2. Selection of Participants

The participants were selected purposively from four upazilas, or administrative sub-districts, Sharishabari, Pirgachha, Lalmohon, and Badarganj, covering three geographical regions (Northern, South-Central, and North-East) in Bangladesh. Twenty-four unions (administrative units consisting of a cluster of villages), six from each upazila, were randomly selected. Subsequently, an approximately equal number of participants was selected from each union using a convenience sampling technique. The selection of the participants is described in detail elsewhere [30].

Female field workers initially surveyed households in the study area to identify eligible study participants based on the date of their last menstrual period (LMP) and history of antenatal clinic (ANC) visits during their current pregnancy. Women were eligible to participate if they had not visited an ANC for a check-up during their current pregnancy.

All potential participants were provided with information about the purpose and the nature of the study before being invited to come to a designated ANC on a pre-set date for data collection. The study protocol was reviewed and approved by the Ethics Committee of the Faculty of Biological Sciences, University of Dhaka, Dhaka, Bangladesh (on 16 April 2015; Ref No. Biol. Sci. 2014–2015).

### 2.3. Data Collection

On the day of data collection, after receiving informed consent from the women, all were tested for confirmation of pregnancy using a commercial pregnancy detection kit. The interviewer reconfirmed the time of their LMP. In total, 530 pregnant women were recruited for the study. The response rate was over 90%.

Structured interviews were conducted by trained interviewers to obtain socio-demographic and pregnancy-related information from the participants. A 7-day food frequency questionnaire (FFQ) was used to gather information on the usual dietary consumption pattern of selected food items rich in micronutrients (red meat (beef, goat, and liver); fish (small and big); dairy (milk and milk products) and eggs; leafy green vegetables; non-leafy vegetables; and seasonal fruits). The FFQ was adopted from the National Micronutrient Survey 2011–2012, modified, and pretested in the study population. Data on the consumption of any vitamin and mineral supplements were collected using a 30-day recall questionnaire. Following the interview, a disposable syringe was used to collect a 5 mL sample of venous blood from each woman. The serum was separated by centrifugation and serum specimens were taken in plastic microcentrifuge tubes and frozen in dry ice, before being transported to a laboratory in Dhaka, and stored at −20 °C until analyzed.

A second blood sample was obtained from the pregnant women after approximately 14 weeks of routine iron (60 mg) and folic acid (400 ug) supplementation per day. Information was also obtained on dietary patterns and consumption of any vitamin or mineral supplements, other than those that were administered during the intervention.

### 2.4. Analytical Procedure

Serum vitamin B_12_ concentrations were measured by electrochemiluminescence immunoassay (ECLIA) on a Roche automated immunoassay analyzer Cobas e601 using a commercial kit, Elecsys Vitamin B12 II (Roche Diagnostics, GmbH, 68305 Mannheim, Germany), according to the manufacturer’s instructions. Preci Control Varia 1 and 2 were used to check both accuracy and precision as an internal quality control material.

### 2.5. Statistical Analysis

Data were analyzed using the statistical software packages IBM SPSS Statistics version 28 (SPSS Inc., Chicago, IL, USA). Due to incomplete data or an insufficient blood sample for vitamin B_12_ assay, eight participants were excluded. Thus, 522 pregnant women were included in the analysis. The distribution of serum vitamin B_12_ concentration was checked by the *Kolmogorov–Smirnov goodness of fit* test and was normally distributed. The univariate analysis comprised a simple frequency distribution of selected variables. Vitamin B_12_ deficiency was defined following the cut-off value suggested by the manufacturer of the kit used in the assay, as serum vitamin B_12_ concentration < 203 pg/mL [31]. Marginal vitamin B_12_ deficiency was defined using the Centers for Disease Control and Prevention (CDC) definition as serum vitamin B_12_ concentration 203 to <300 pg/mL [32].

A paired *t*-test was applied to compare the difference of mean serum vitamin B_12_ concentrations between women during early (at baseline) and late pregnancies (when they had the second blood sample; after 14 weeks of IFA supplementation). The differences in the prevalence of vitamin B_12_ deficiency and marginal deficiency between early and late pregnancies were examined using a chi-squared test. The differences in marginal vitamin B_12_ status and deficiency in late pregnancy were also compared between groups of various socio-demographic, pregnancy, and diet-related characteristics using a chi-squared test.

Finally, a logistic regression analysis was used to determine the independent association of selected socio-demographic, pregnancy, and diet-related variables with marginal vitamin B_12_ status and vitamin B_12_ deficiency separately among women during late pregnancy. The independent variables included in the analysis were age, parity, gestational age, participants, and their husband’s education level and occupation, household size, home ownership, cultivable land ownership, taking vitamin and mineral supplements, and consumption of red or organ meat, fish, eggs, and dairy products. The odds ratio (OR) and 95% confidence interval (CI) were calculated. The *p*-value ˂ 0.05 was considered statistically significant.

## 3. Results

A total of 522 participants enrolled during early pregnancy, and 405 completed the study protocol of 14 weeks follow up. Thus, the drop-out rate was 22.4%. There were no significant differences between the various socio-economic and pregnancy-related characteristics of the pregnant women who completed the study and those who did not (data not shown).

At the time of recruitment, the age of the participants ranged from 13–38 years (mean ± SD age 23.6 ± 4.8 years) and gestational age (GA) ranged from 7 to 20 weeks (mean ± SD GA 15.2 ± 2.7 weeks). A large majority of the participants (44%) and their husbands (57.5%) were functionally illiterate (had never been to school or had completed up to grade 5 only). Ninety-six percent of the participants were homemakers and about two-thirds of their husbands were either day laborers or farmers. Nine out of 10 participants owned their own homes, while over half (56.5%) of the participants had no cultivable land (Table 1).

The distributions of the participants by age, parity, and gestational age group between early and late pregnancies were not significantly different (Table 1). Similarly, the distributions of the participants by various socio-economic groups did not differ significantly between early and late pregnancies (Table 1). The mean serum vitamin B_12_ concentration during late pregnancy was significantly lower (*p*-value = 0.0001) than during early pregnancy (Table 2). The mean (SD) difference of serum vitamin B_12_ concentration between women in their early and late pregnancies was −56.2 (65.7) pg/mL.

**Figure 1:**
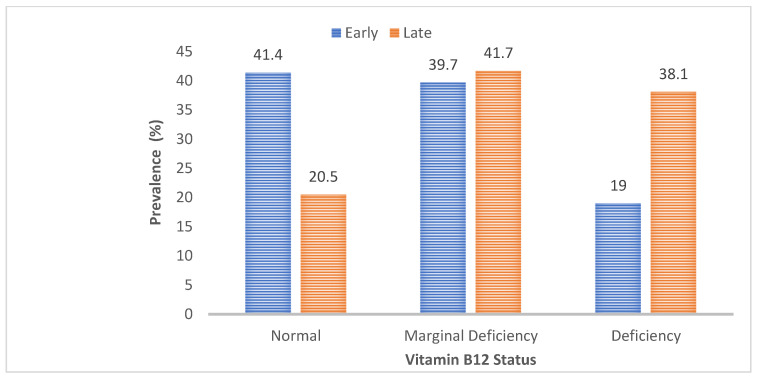
Prevalence of vitamin B_12_ deficiency (serum vitamin B_12_ concentration < 203 pg/mL) and marginal deficiency (serum vitamin B_12_ concentration 203 to <300 pg/mL) during early and late stages of pregnancy among rural Bangladeshi women.

Figure 1 shows the prevalence of vitamin B_12_ deficiency and marginal deficiency with the progress of pregnancy. During the early pregnancy stage, nearly 40% of the women had a marginal vitamin B_12_ status (serum vitamin B_12_ concentration 203 to <300 pg/mL) and 19% had vitamin B_12_ deficiency (serum vitamin B_12_ concentration < 203 pg/mL). After 14 weeks of follow up, during the late pregnancy stage (mean ± SD GA 29 ± 2.6 weeks), the prevalence of marginal vitamin B_12_ deficiency remained unchanged (41.7%), whereas vitamin B_12_ deficiency rose significantly to 38%.

For the selected food items rich in vitamin B_12_, the distribution according to the frequency of consumption by women during their late pregnancy is shown in Table 3. Nearly 42% percent of the women did not consume red meat or organ meat at all in the 7 days preceding the interview, and another 32% of the women had it only once or twice a week. A large majority of the pregnant women (46%) had milk seven times or more per week; however, one in four women (23.5%) did not consume milk at all. About 30% of the women had eggs seven times or more per week, whereas 19% of the women did not consume eggs at all. It was also noted that one in four women (24.9%) did not consume fish at all.

Table 4 illustrates the differences in the prevalence of vitamin B_12_ deficiency and marginal deficiency during late pregnancy by various socio-demographic, dietary, and pregnancy-related factors. Using bivariate analysis, the prevalence of both vitamin B_12_ deficiency and marginal vitamin B_12_ deficiency was higher among pregnant women with a gestational age of ≥27 weeks than in the pregnant women with a gestational age of <27 weeks. However, the difference was not statistically significant (*p* = 0.081). The prevalence of vitamin B_12_ deficiency was significantly higher (*p* = 0.005) among pregnant women who lived in a rented house than in the pregnant women who owned their own house. The prevalence of vitamin B_12_ deficiency and/or marginal deficiency during late pregnancy was not influenced by any other socio-demographic or pregnancy-related factors. The prevalence of vitamin B_12_ deficiency was significantly higher (*p* = 0.019) among pregnant women who consumed red or organ meat fewer than three times a week than in the pregnant women who consumed such meat three times or more per week.

The factors associated with vitamin B_12_ deficiency and marginal deficiency were separately examined using logistic regression analysis (Table 5). The pregnant women with a gestational age of ≥27 weeks had a higher risk of developing marginal vitamin B_12_ deficiency (adjusted OR = 1.98; 95% CI = 0.904–4.342; *p* = 0.088) and vitamin B_12_ deficiency (adjusted OR = 2.61; 95% CI = 1.096–6.214; *p* = 0.03) than the pregnant women with a gestational age of <27 weeks. The pregnant women with a parity of two or more were 2.74 times more likely to suffer from vitamin B_12_ deficiency than the nullipara pregnant women (adjusted OR = 2.744; 95% CI = 0.918–8.204; *p* = 0.07). The risk of vitamin B_12_ deficiency was significantly higher in pregnant women who did not have their own house (adjusted OR = 13.32; 95% CI = 1.55–114.25; *p* = 0.018). The pregnant women who usually consumed red or organ meat < 3 times a week were 2.33 times more likely to suffer from vitamin B_12_ deficiency compared with the pregnant women who consumed red or organ meat > 3 times a week (adjusted OR = 2.327, 95% CI = 1.194–4.536; *p* = 0.013). However, the risk of marginal vitamin B_12_ deficiency was only 1.69 times higher among pregnant women who ate red or organ meat < 3 times a week (adjusted OR = 1.689, 95% CI = 0.908–3.140; *p* = 0.098).

## 4. Discussion

This is first study that reports the changes in the prevalence of vitamin B_12_ deficiency with the progress of pregnancy, and the factors associated with vitamin B_12_ deficiency and marginal deficiency among pregnant women in rural Bangladesh. The majority of the participants in this study were functionally illiterate; they were homemakers who came from a low socio-economic background.

The study revealed that the prevalence of vitamin B_12_ deficiency among these women increased significantly with the progress of their pregnancies. During early pregnancy, 19% of the women had vitamin B_12_ deficiency, which doubled to 38% during late pregnancy. However, the prevalence of marginal vitamin B_12_ deficiency remained unchanged. This could indicate that, as more women who were marginally deficient became deficient with the progression of pregnancy, there were additional women with B_12_ sufficiency who gradually became marginally deficient, thus keeping the prevalence rate similar.

While there are limited data on vitamin B_12_ deficiency and marginal deficiency among pregnant Bangladeshi women, one study conducted in rural north-western Bangladesh reported a 20% prevalence of vitamin B_12_ deficiency during early (median GA of 10 weeks) pregnancy [26]. A randomized controlled trial with a small sample size conducted in Dhaka City reported a 26% prevalence of vitamin B_12_ deficiency and another 40% with marginal vitamin B_12_ deficiency during the early stage (GA of 11–14 weeks) of pregnancy [28]. Thus, the prevalence of vitamin B_12_ deficiency in our study population was comparable to that observed in these two studies. However, an earlier study by Lindström et al. conducted in a sub-district of rural Bangladesh reported a 46% prevalence of vitamin B_12_ deficiency during early pregnancy [27], which was more than double that in the present study. There can be several reasons for the differences in the prevalence of vitamin B_12_ deficiency between the present study and the research conducted by Lindström et al. For instance, Lindström et al.’s study was conducted in only one sub-district of rural Bangladesh, while our study included pregnant women from four sub-districts from different geographical regions in rural Bangladesh. Furthermore, the study by Lindström et al. was conducted almost 18 years ago and did not reflect the present scenario. Similar to the situation in many resource poor countries, a low intake of animal source food is a major cause of poor vitamin B_12_ status in Bangladesh. The available data indicate that there has been an overall increase in the consumption of animal foods in the country (26.2 g/capita per d in 2010 v. 20.8 g/capita per d in 2005) [33], which might have contributed to a variation in the prevalence of vitamin B_12_ deficiency. In addition, Lindström et al. used radioimmunoassay (RIA) for the assessment of vitamin B_12_ status, while we used electrochemiluminescence immunoassay, which is a more sensitive, reliable, and advanced method than RIA [34]. Thus, the methodological differences in the assessment of vitamin B_12_ status between the two studies may also explain the differences in the prevalence of vitamin B_12_ deficiency.

The present study reveals a significant increase in the prevalence of vitamin B_12_ deficiency with the progress of pregnancy. A study carried out among pregnant women in Venezuela reported a 50% prevalence of vitamin B_12_ deficiency in the first trimester, 59% in the second trimester, and 72.5% in the third trimester; the authors concluded that the prevalence of vitamin B_12_ deficiency rises as pregnancy advances [35]. Another study conducted in Canada showed a 35% prevalence of vitamin B_12_ deficiency during early pregnancy; as the pregnancy advanced, there was a significant rise in the prevalence of vitamin B_12_ deficiency to 42.9% [36]. A systematic review, based on worldwide pooled trimester-wise estimates, reported a steady increase in the prevalence of vitamin B_12_ deficiency with the progress of pregnancy [4]. Thus, the findings in the present study are consistent with the findings of previous studies.

Studies from the Netherlands [37], Spain [10], Canada [36], India [5], and in 12 out of 13 longitudinal studies included in a systematic review [4] reported a significant decline in the serum concentration of vitamin B_12_ as a pregnancy progressed. The present study also found a significant decline in the mean serum vitamin B_12_ concentration (*p* = 0.0001) from the early to late stages of pregnancy, where the mean concentration during early pregnancy was 300 pg/mL; with the advancement of pregnancy, the serum concentration fell by 56 pg/mL (19% decrease) during the late stage of pregnancy. There could be several possible reasons for the gradual decrease in serum vitamin B_12_ concentration during pregnancy. For example, alterations in the concentration of vitamin B_12_ binding proteins [38]. Grebe et al. [38], in their study, showed that the decline in serum vitamin B_12_ concentration was closely related to the decline in the fraction of B_12_ bound to haptocorrin (holo-haptocorrin) during pregnancy, while the fraction of B_12_ bound to transcobalamin (holo-transcobalamin) remained unchanged. Furthermore, there were no changes in the concentration of vitamin B_12_ analogs bound to haptocorrin during pregnancy. The other reasons could be due to increased maternal nutritional and physiological demands as the pregnancy progressed, hemodilution due to plasma volume expansion, hormonal changes, and/or increased placental transfer of vitamin B_12_ to the fetus [39,40,41]. In addition, low dietary intake of vitamin B_12_ or lack of access to B_12_ fortified foods or B_12_ supplements further precipitates the decline in B_12_ and could be an important but modifiable cause of poor vitamin B_12_ status [21,23,41].

While we do not have quantitative estimates of dietary intake of vitamin B_12_, the present study collected data on the frequency of intake of selected animal source food rich in vitamin B_12_. Two in five women (41.7%) reported not consuming red or organ meat at all over a period of one week preceding the interview. Nearly a quarter of the women never had fish, eggs, or milk. Furthermore, nearly a third of the pregnant women had red meat, fish, and eggs only 1 to 2 times per week. Of note, vitamin B_12_ fortified foods are not generally available in Bangladesh. In addition, none of the participants reported taking vitamin B_12_ supplements. Therefore, it is highly likely that low dietary intake of vitamin B_12_ might have contributed to the poor vitamin B_12_ status in this population. Siddiqua et al. [28] in their study among pregnant women in Bangladesh also reported limited intake of animal-source food. A study by Herrán et al. [23] also reported a low intake of animal-source foods in a Colombian population with a high prevalence of vitamin B_12_ deficiency.

The present study explored the association of vitamin B_12_ deficiency and marginal vitamin B_12_ deficiency with various socio-economic, pregnancy, and diet-related factors. The results of bivariate analysis reveal a significantly higher prevalence of vitamin B_12_ deficiency among pregnant women who lived in a rented house (*p* = 0.005) and those who consumed red or organ meat fewer than three times per week (*p* = 0.019).

We conducted separate logistic regression analysis to identify the factors that were independently associated with vitamin B_12_ deficiency and marginal vitamin B_12_ deficiency during late pregnancy by taking into account of potential confounders. We found that the women with a gestational age of ≥27 weeks had a 2.6 times higher risk of becoming vitamin B_12_ deficient than the women with a gestational age of <27 weeks. While the risk of marginal vitamin B_12_ deficiency in women with a gestational age of ≥27 weeks was nearly double that of women with a gestational age of <27 weeks, the difference was not statistically significant. Sukumar et al. [4], in their systematic review and meta-analysis of the prevalence of vitamin B_12_ insufficiency in pregnancy examining worldwide pooled trimester wise estimates, also reported an increased prevalence of vitamin B_12_ insufficiency with the increase in gestational age, thus supporting our findings. On the contrary, Barney et al. [42] conducted a study among pregnant rural South Indian women and reported nearly four-times higher odds of being B_12_ deficient for women in the first trimester compared with those in the second trimester. The authors mentioned that the increased odds of vitamin B_12_ deficiency in the first trimester could be due to decreased intake because of morning sickness.

Although not statistically significant (*p* = 0.07), women with parity of two or more had a 2.74 times higher odds of being vitamin B_12_ deficient compared to nullipara pregnant women. On the contrary, a study conducted in South India reported that primipara women had a 1.4 times higher risk of developing impaired vitamin B_12_ status defined by low serum B_12_ concentration and elevated methyl malonic acid [43]. Another study conducted among pregnant women in Amsterdam showed that nulliparous women had a significantly lower concentration of vitamin B_12_ compared with multiparous women and concluded that nulliparous women were more at risk of developing vitamin B_12_ deficiency [44]. The discrepancy between the findings of the previous studies and present study could be due to the differences in pre-pregnancy vitamin B_12_ status and/or dietary intake of vitamin B_12_ during pregnancy.

The present study failed to show any association between various socio-economic factors and the risk of vitamin B_12_ deficiency, except for home ownership. The logistic regression revealed that the odds of developing vitamin B_12_ deficiency were 13 times higher among pregnant women who lived in rented accommodation compared with those who lived in their own house. A study conducted among infants in Nepal also showed that families that lived in their own house had a higher concentration of serum vitamin B_12_ than those living in a rented house [45]. A study conducted among Colombian women also found no association between socio-economic status and serum vitamin B_12_ deficiency [46]. Another study of pregnant Colombian women reported a positive association between the education level of the household head and serum vitamin B_12_ concentrations, but could not find any association with wealth index and/or food security [23].

The present study also found that the risk of vitamin B_12_ deficiency was 2.33 times higher among women who consumed red or organ meat <3 times a week. While not statistically significant (*p* = 0.098), the risk of marginal vitamin B_12_ deficiency in women who consumed red or organ meat <3 times a week was 1.68 times higher. A study conducted among pregnant women in South India examining the relationship between consumption of food rich in vitamin B_12_ reported that the participants who consumed fish and yogurt more frequently had a higher concentration of serum vitamin B_12_ and were less likely to develop a vitamin B_12_ deficiency [43]. Another study among Dutch women during late pregnancy showed that vitamin B_12_ intake from dairy, meat and fish, but not eggs was independently associated with plasma concentrations of total vitamin B_12_ in a dose response manner. Furthermore, the intake of these foods was also independently associated with reduced odds of vitamin B_12_ deficiency [47]. One of the reasons for the discrepancy between previous studies and our study could be due to the variations in the amount of vitamin B_12_ intake from various animal source foods. Of note, in the present study, we collected data on the frequency of consumption, but without the portion size; thus, we were unable to determine the actual amount of vitamin B_12_ intake from each of these animal source foods.

The strength of this study is that it represents a relatively large sample from different geographical areas in rural Bangladesh, and, for the first time, employed a longitudinal study design to assess the changes in the prevalence of vitamin B_12_ deficiency with the progress of a pregnancy. However, this study also has some limitations. First, we used a convenience sampling method for selecting the study participants, and therefore the findings of this study may not be representative of the wider population from which the participants were drawn. Second, the dietary data focused on the frequency of consumption of various animal source foods, but not the specific amount of consumption. A more in-depth dietary assessment, including a quantitative estimation of vitamin B_12_ intake, should be considered in future studies. Third, although serum vitamin B_12_ concentrations are a commonly used biomarker for assessing vitamin B_12_ status in population-based studies [22], it is not a reliable indicator of vitamin B_12_ status during pregnancy. Thus, the findings of the study should be interpreted with caution. Other biomarkers, such as circulating holo-transcobalamin (refer to as active vitamin B_12_) and methylmalonic acid (MMA, functional biomarker), are more sensitive indicators of vitamin B_12_ status during pregnancy than serum vitamin B_12_ concentration [3,13]. Further, circulating holo-transcobalamin concentrations remain relatively unchanged during pregnancy [3,38] and it is a more sensitive indicator of vitamin B_12_ status than the serum MMA concentration [48]. Future studies should include other markers, such as circulating holo-transcobalamin, which would enhance the accuracy in the assessment of vitamin B_12_ status during pregnancy, and thus the interpretation of the findings.

## 5. Conclusions

In conclusion, we found a high prevalence of vitamin B_12_ deficiency and marginal deficiency among pregnant rural women in Bangladesh, with a significant increase in the prevalence of vitamin B_12_ deficiency with the progress of pregnancy. The finding raises concerns as it could impact on pregnancy outcomes. Furthermore, increasing gestational age, higher parity, living in a rented house, and consumption of red or organ meat fewer than three times a week were identified as potential risk factors of vitamin B_12_ deficiency in this population. Further research should focus on a more in-depth assessment of dietary vitamin B_12_ intakes, along with identifying other non-dietary risk factors of vitamin B_12_ deficiency, to develop an appropriate intervention program to prevent vitamin B_12_ deficiency in this population.

## Figures and Tables

**Table 1:** Socio-demographic and pregnancy-related characteristics of the women by early and late stages of pregnancy.

Variable	Early Pregnancy (*n* = 522) (7–20 Weeks)		Late Pregnancy (*n* = 404) (21–34 Weeks)	
	*n*	%	*n*	%
Age (Year)				
Adolescent (13–19)	126	24.1	97	24.0
Young adult (20–24)	166	31.8	126	31.2
Young adult2 (>25)	230	44.1	181	44.8
Gestational Age (Week) *				
<13	71	13.6	49	12.1
13 or more	451	86.4	355	87.9
Parity				
No living child	197	37.7	151	37.4
Only one	205	39.3	158	39.1
Two or more	120	23.0	95	23.5
Paricipant’s Education				
Functionally illiterate **	230	44.1	174	43.1
Grade 6 to 9	203	38.9	166	41.1
SSC or above	89	17.0	64	15.8
Husband’s Education				
Functionally illiterate	300	57.5	234	57.9
Grade 6 to 9	111	21.3	91	22.5
SSC or above	111	21.3	79	19.6
Husband’s Occupation				
Day laborer	205	39.3	151	37.4
Farmer	136	26.1	115	28.5
Business/service	181	34.6	138	34.1
Participant’s Occupation				
Homemaker	502	96.2	387	95.8
Working	20	3.8	17	4.2
Family Size				
Small family (up to 4)	315	60.3	239	59.2
Large family (5 or more)	207	39.7	165	40.8
Home Owner				
No	38	7.3	25	6.2
Yes	484	92.7	379	93.8
Cultivable Land Ownership				
No land	295	56.5	228	56.4
Small landholding	227	43.5	176	43.6

* Gestational age at recruitment. ** No formal education or studies less than grade 5. Abbreviations: SSC—Secondary School Certificate.

**Table 2:** Mean difference in serum vitamin B_12_ concentrations between women in their early and late pregnancies.

Variables	*n*	Mean (pg/mL)	SD	*p*-Value *
Early pregnancy (7–20 weeks)	522	299.9	121.1	0.0001
Late pregnancy (21–34 weeks)	404	243.8	92.8	
Mean changes (late–early)	404	−56.2	65.7	

* Paired *t*-test.

**Table 3:** Frequency of consumption of various foods rich in vitamin B_12_ by the pregnant women during late stage of pregnancy.

	Never		1–2 Times/Week		3–4 Times/Week		5–6 Times/Week		≥7 Times/Week	
	*n*	%	*n*	%	*n*	%	*n*	%	*n*	%
Eggs	77	19.0	113	27.9	75	18.5	19	4.7	121	29.9
Milk	95	23.5	60	14.8	39	9.6	24	5.9	187	46.2
Meat	169	41.7	130	32.1	65	16.0	25	6.2	16	4.0
Fish	101	24.9	129	31.9	84	20.7	35	8.7	46	13.8

**Table 4:** Prevalence of vitamin B_12_ deficiency and marginal deficiency during late pregnancy by socio-demographic, dietary, and pregnancy-related factors.

		Normal		Marginally Deficient		Deficiency		
		*n*	%	*n*	%	*n*	%	*p*-Value *
Age (Year)	Total (*n*)							0.672
Adolescent (13–19)	97	18	18.6	46	47.4	33	34.0	
Young adult (20–24)	126	28	22.2	47	37.3	51	40.5	
Young adult2 (>25)	181	37	20.4	74	40.9	70	38.7	
Gestational Age (Week)								0.081
<27	49	16	32.7	18	36.7	15	30.6	
27 or more	355	67	18.9	149	42.0	139	39.2	
Parity								0.120
No living child	151	29	19.2	72	47.7	50	33.1	
Only one	158	38	24.1	61	38.6	59	37.3	
Two or more	95	16	16.8	34	35.8	45	47.4	
Paricipant’s Education								0.805
Functionally illiterate	174	36	20.7	70	40.2	68	39.1	
Grade 6 to 9	166	32	19.3	74	44.6	60	36.1	
SSC or above	64	15	23.4	23	35.9	26	40.6	
Husband’s Education								0.655
Functionally illiterate	234	43	18.4	102	43.6	89	38.0	
Grade 6 to 9	91	21	23.1	33	36.3	37	40.7	
SSC or above	79	19	24.1	32	40.5	28	35.4	
Husband’s Occupation								0.685
Day laborer	151	31	20.5	56	37.1	64	42.4	
Farmer	115	23	20.0	50	43.5	42	36.5	
Business/service	138	29	21.0	61	44.2	48	34.8	
Participant’s Occupation								0.591 **
Homemaker	387	81	20.9	158	40.8	148	38.3	
Working	17	2	11.8	9	52.9	6	35.3	
Family Size								0.956
Small family (up to 4)	239	48	20.1	99	41.4	92	38.5	
Large family (5 or more)	165	35	21.2	68	41.2	62	37.6	
Home Owner								0.005 **
No	25	1	4.0	7	28.0	17	68.0	
Yes	379	82	21.6	160	42.2	137	36.1	
Cultivable Land Ownership								0.631
No land	228	45	19.7	99	43.4	84	36.8	
Small landholding	176	38	21.6	68	38.6	70	39.8	
Meat Intake (Frequency/Week) ***								0.019
<3	299	53	17.7	122	40.8	124	41.5	
3 times or more	105	30	28.6	45	42.9	30	28.6	
Fish Intake (Frequency/Week)								0.653
<3	230	51	22.2	93	40.4	86	37.4	
3 times or more	174	32	18.4	74	42.5	68	39.1	
Milk Intake (Frequency/Week)								0.871
<3	155	30	19.4	64	41.3	61	39.4	
3 times or more	249	53	21.3	103	41.4	93	37.3	
Eggs (Frequency/Week)								0.688
<3	190	36	18.9	78	41.1	76	40.0	
3 times or more	214	47	22.0	89	42.6	78	36.4	
Vitamin/Mineral Supplement								0.742
No	376	76	20.2	155	41.2	145	38.6	
Yes	28	7	25.0	12	42.9	9	32.1	

* Chi-squared test ** Exact test. *** Red or organ meat. Abbreviations: SSC—Secondary School Certificate.

**Table 5:** Logistic regression analysis for odds of vitamin B_12_ deficiency and marginal deficiency by various factors among rural Bangladeshi women during late pregnancy.

Variable	Marginally Deficient				Vitamin B_12_ Deficiency			
	Exp (B)	95% CI for EXP(B)		*p-*Value	Exp (B)	95% CI for EXP(B)		*p-*Value
		Lower	Upper			Lower	Upper	
Age (Year)								
Adolescent (13–19) (Ref Cat **)	1.0				1.0			
Young adult (20–24)	0.929	0.399	2.164	0.864	0.716	0.276	1.859	0.492
Young adult2 (>25)	1.028	0.371	2.852	0.957	0.490	0.162	1.483	0.207
Gestational Age (Week)								
<27 (Ref Cat)	1.0				1.0			
27 or more	1.981	0.904	4.342	0.088	2.610	1.096	6.214	0.030
Parity								
No living child (Ref Cat)	1.0				1.0			
Only one	0.598	0.264	1.356	0.218	1.397	0.576	3.305	0.471
Two or more	0.734	0.255	2.112	0.566	2.744	0.918	8.204	0.071
Participant’s Education								
Functionally illiterate	0.911	0.301	2.759	0.870	0.519	0.171	1.572	0.246
Grade 6 to 9	1.408	0.549	3.614	0.477	0.667	0.254	1.756	0.413
SSC or above (Ref Cat)	1.0				1.0			
Husband’s Education								
Functionally illiterate	1.723	0.648	4.579	0.275	1.799	0.651	4.967	0.257
Grade 6 to 9	0.976	0.394	2.417	0.958	1.559	0.577	4.214	0.381
SSC or above (Ref Cat)	1.0				1.0			
Husband’s Occupation								
Day laborer	0.682	0.332	1.402	0.298	1.003	0.468	2.150	0.993
Farmer	0.934	0.443	1.969	0.857	0.864	0.388	1.924	0.721
Business/service (Ref Cat)	1.0				1.0			
Family Size								
Small family (up to 4) (Ref Cat)	1.0				1.0			
Large family (5 or more)	0.875	0.477	1.604	0.665	1.009	0.531	1.919	0.978
Home Owner								
No	3.158	0.340	29.340	0.312	13.32	1.55	114.25	0.018
Yes (Ref Cat)	1.0				1.0			
Cultivable Land Ownership								
No land	1.367	0.702	2.659	0.358	1.223	0.623	2.400	0.558
Sizeable land holding (Ref Cat)	1.0				1.0			
Meat * Intake								
<3 times	1.689	0.908	3.140	0.098	2.327	1.194	4.536	0.013
3 times or more (Ref Cat)	1.0				1.0			
Fish Intake								
<3	0.640	0.357	1.147	0.134	0.628	0.339	1.162	0.138
3 times or more (Ref Cat)	1.0				1.0			
Milk Intake								
<3 times	0.948	0.509	1.764	0.866	0.919	0.482	1.754	0.798
3 times or more (Ref Cat)	1.0				1.0			
Egg Intake								
<3 times	1.138	0.635	2.041	0.664	1.235	0.664	2.297	0.506
3 times or more (Ref Cat)					1.0			

* Red or organ meat. ** Reference Category. Abbreviations: SSC—Secondary School Certificate.

## Data Availability

The data presented in this study are available on request from the corresponding author.
